# Hydrophosphonylation of Nanoparticle Schiff Bases as a Mean for Preparation of Aminophosphonate-Functionalized Nanoparticles

**DOI:** 10.3390/molecules18078473

**Published:** 2013-07-18

**Authors:** Justyna Siemieniec, Pawel Kafarski, Pawel Plucinski

**Affiliations:** 1 Department of Bioorganic Chemistry, Faculty of Chemistry, Wrocław University of Technology, Wybrzeże Wyspiańskiego 27, 50-370 Wroclaw, Poland; 2 Department of Chemical Engineering, University of Bath, Bath BA2 7AY, UK

**Keywords:** magnetic nanoparticles, aminophosphonates, surface modification

## Abstract

The development of nanotechnology is responsible for an increase in the achievements in medical diagnostics and in the preparation of new therapeutic vehicles. In particular, magnetic nanoparticles with a modified surface are a very attractive alternative to deliver therapeutic agents. We describe the modification of the surface of the iron oxide nanoparticles with aminophosphonic acids by applying the classic hydrophosphonylation approach.

## 1. Introduction

The dynamic development of nanotechnology (including nanoparticle technology) and molecular biology is the cause of a rapid increase in the achievements in medical diagnostics and in the preparation of new therapeutic substances [1,2,3]. Nanoparticles with a suitably chemically modified surface may be used for many applications, such as magnetic resonance imaging contrast enhancement, immunoassay, detoxification of biological fluids, drug delivery devices, and in cell separation [4,5]. Magnetic nanovehicles are especially very attractive for the delivery of therapeutic agents. They can be targeted to specific locations in the body through the application of an external magnetic field gradient, as has been documented by a number of promising animal and clinical trials [6,7,8].

In this paper we describe the usefulness of hydro-phosphonylation of the C=N bond for the modification of the surface of the iron oxide nanoparticles with aminophosphonic acids. Such aminophosphonate nanoparticles should act as interesting, surfactant-like species because depending on the pH of the solution, they may appear as negatively (because of the presence of phosphonate groups in their structure), positively (because of the presence of ammonium groups) or zwitterionic species, thus being able to exhibit non-typical properties, similar to those of recently obtained gold-supported negatively charged surfactant-like nanoparticles [9]. This property should facilitate their ability to bind organic compounds, proteins and metal ions. 

The combination of the biological properties of aminophosphonates [10,11] and the potential of the magnetic nanoparticles for effective targeted delivery using an external magnetic force, should lead to magnetic bio-conjugates for selective targeting of physiologically active aminophosphonates to organs which require their activity. The nanoparticles covered with aminophosphonates may also be used as the supports for the immobilization of other drugs (especially anticancer ones) or for the immobilization of enzymes. Additionally, they can be considered as a system for the removal of metal ions from environmental and body fluids [12]. This could be achieved because aminophosphonic acids are bifunctional molecules and possess two reactive moieties—amino and phosphonate groups. Thus, when present at the surface of nanoparticles they could be used to bind other substances (including proteins) either covalently or by adsorption. This property should facilitate their ability to bind organic compounds, proteins and metal ions. In this paper we describe our preliminary studies on the modification of superparamagnetic iron oxide nanoparticles with aminophosphonic acids

## 2. Results and Discussion

### 2.1. Preparation of Modified Nanoparticles

Iron oxide nanoparticles obtained by the standard co-precipitation method from iron(II) and iron(III) chlorides in the presence of ammonium hydroxide solution [13], were coated with tetraethyl orthosilicate and their surface was further functionalized with (3-aminopropyl)triethoxysilane (APTMS) [14].

**Figure 1:**
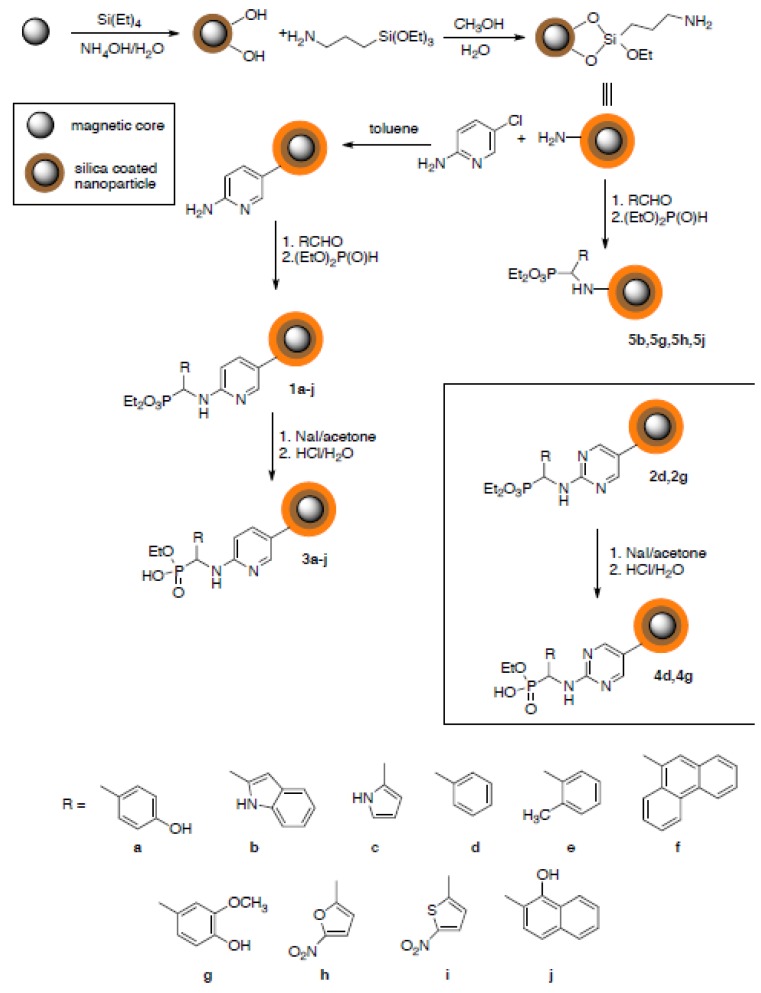
Synthetic scheme leading to aminophosphonate-functionalized magnetic nanoparticles.

Magnetic iron oxide nanoparticles coated with structurally variable aminophosphonates have been obtained by hydrophosphonylation of Schiff bases. We have applied its reacted version commonly used in aminophosphonate synthesis [15]. The direct application of this procedure when (3-aminopropyl)-triethoxysilane functionalized nanoparticles were used gave unsatisfactory results, because of strong aggregation of the resulting nanoparticles. This might be due to the high density of amino groups on the nanoparticles’ surface and their simultaneous low flexibility. Therefore, we decided to apply the double-functionalization approach using aminopyridine or diaminopyrimidine as linkers. The synthetic procedure, schematically shown in Figure 1, provided two series of nanoparticles **1a**–**j** and **2d** and **2g**. Finally, one phosphonate moiety was hydrolyzed with sodium iodide in acetone followed by acidification with 1M hydrochloric acid, a reaction which provided nanoparticles functionalized with aminophosphonate esters. Finally, one phosphonate moiety was desterilized with sodium iodide in acetone followed by acidification with 1M hydrochloric acid, a reaction which provided nanoparticles functionalized with aminophosphonate esters. Hydrolysis to monoester is of strategic importance—in further studies, the resulting compound library will be used to further immobilization, including enzymes.

### 2.2. Characterization of Nanoparticles

**Figure 2:**
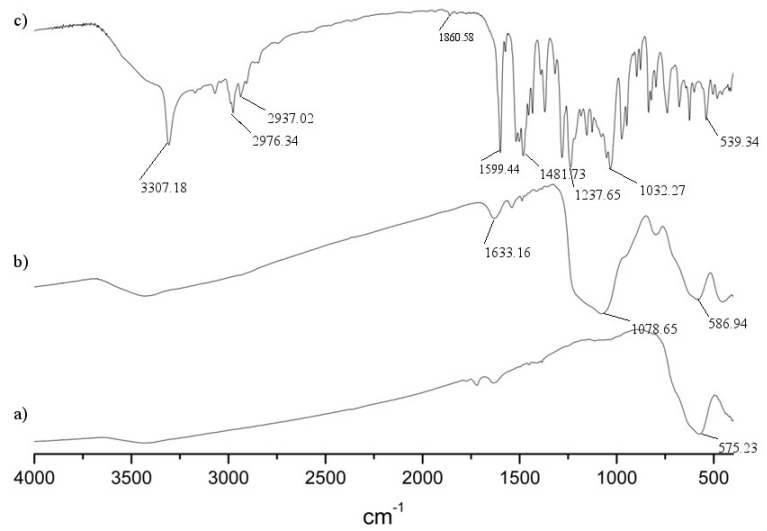
IR spectra of (**a**) iron oxide nanoparticle; (**b**) nanoparticle coated with silica and functionalized with APTMS; (**c**) aminophosphonate-functionalized nanoparticle **1g**.

The formation of functionalized nanoparticles is accompanied by the sharpening of the IR spectra, which is in good accordance with the literature [16,17]. The infrared spectra of unmodified and aminophosphonate-modified iron oxide nanoparticles are shown in Figure 2. The IR spectrum of pure iron oxide nanoparticle shows a strong and wide band around 580 cm^−1^, characteristic for the Fe–O stretching vibrations related to the magnetic core [Figure 2(a)]. A broad peak at around 1,078 cm^−1^ corresponds to the Si-O and Si-C antisymmetric out of plane stretching bands, which are overlapping [Figure 2(b)]. Binding of aminophosphonate results in significant changes in the spectra [Figure 2(c)]. They are similar to those which are expected for aminophosphonates. The presence of P=O results in the stretching band at 1,230 cm^−1^, whereas the peak at 1,030 cm^−1^ corresponds to both P-O-C and Ar-O-C vibrations (the latter one in the particular cases of particles **1g** and **1j**). The peak at 1,600 cm^−1^ most likely corresponds to the N-H bending scissoring vibrations, while C-N bond stretching is seen at 1,480 cm^−1^.

**Figure 3:**
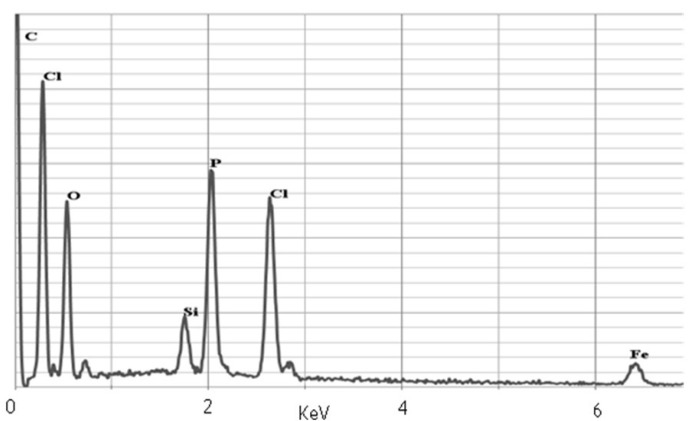
EDX spectrum of **1g** nanoparticles.

In order to determine if there is a phosphorus atom present at the surface of the obtained nanoparticles we have applied energy-dispersive X-ray spectroscopy (SEM-EDX), which gave expected results (Figure 3). During sample scanning the obtained bands clearly showed the presence of the following atoms: Fe (6.5 and 0.8 KeV), Si (1.9 KeV), P (2.0KeV), O (0.5KeV) and C (0.1 KeV). Intensified signals at 0.1, 0.3, 2.5 are derived from elements contained in the matrix: C (0.1 KeV) and Cl (0.3 KeV, 2.5 KeV).

Due to the magnetic properties of iron oxide nanoparticles we could not apply ^31^P-NMR, a technique typically used for the determination of the presence of carbon-to-phosphorus bonds. Therefore we have decided to obtain pure silica oxide nanoparticles and functionalize their surface with representative aminophosphonates in a manner identical to the magnetic ones. We expected that the infrared spectra of these nanoparticles were going to be similar to those obtained for functionalized magnetic nanoparticles showing only the lack of the band corresponding to the Fe-O vibrations. 

**Figure 4:**
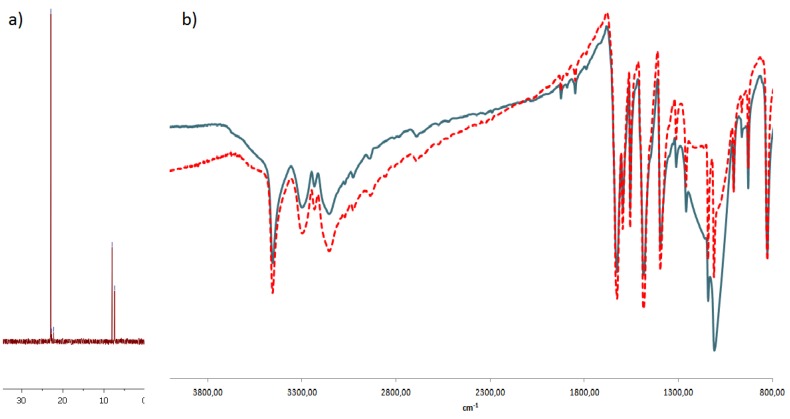
(**a**) ^31^P-NMR spectrum of nanoparticle **1g** lacking magnetic core (**b**) Comparison of IR spectrum with magnetic core (blue line) and without (red line) of nanoparticles modified with 2,6-diamino-4-chloropyrimidine.

On the other hand, the fact that this system is not magnetic allows us to perform ^31^P-NMR measurements. The silica nanoparticles, lacking the iron oxide magnetic core, were synthesized from tetraethylortosilicate following the procedure described in the literature. In the next step we modified their surfaces with (3-aminopropyl)-trimethoxysilane and then with aminophosphonates following the procedure elaborated in this work. The IR spectra received for these particles, lacking the magnetic core, were identical to those obtained for magnetic ones. The ^31^P-NMR spectra obtained for the nanoparticles clearly indicated the presence of a phosphonate moiety appearing at about 20 ppm, which corresponded to the expected products (Figure 4). This finding unequivocally confirms that the procedure described in this paper results in iron oxide nanoparticles surface covered with aminophosphonates. Between 5 and 10 ppm additional peaks corresponding to diethyl and monoethyl phosphites could be observed. Their appearance results from the fact that we had not purified the functionalized silica.

### 2.3. Nanoparticles Size and Dispersion

**Figure 5:**
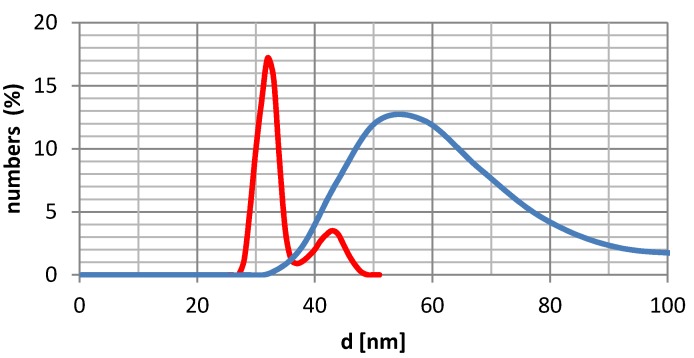
DLS measurements of iron oxide nanoparticles (red line) and with silica core (blue line).

The size and distribution of oxide nanoparticles have been measured using dynamic light scattering. The size distribution of non-functionalized iron oxide nanoparticles, is between 15–50 nm, with a maximum value at 32 nm (Figure 5). The size distribution of functionalized particles clearly shows that they have a strong tendency to agglomerate. This is clearly seen from the transmission electron microscopy measurements (TEM), which support both the nearly uniformly sized uncoated iron oxide and the wide distribution of the size of the functionalized ones results from aggregation. The degree of aggregation is dependent on the structure of nanoparticle coating (presence or absence of linker), the structure of the aminophosphonate side chain and on the solvent used (measurements were done in water, methanol, toluene and acetone). The measurements indicated that the average diameter of the obtained nanoparticles appeared to be the smallest in methanol, with compounds **1a** **(**hydrodynamic size 144 nm), **1c** (72 nm), **1f** (70 nm), **1i** (65 nm) and **1j** (139nm) being representative examples. The respective acids **1a**, **1c**, **1d**, **4d** and **4g** also exhibit low size of dispersion of 50–200 nm. 

The measurements in acetone also gave satisfactory results—some nanoparticles (**1f**, **1c** and **5b**) exhibit a low range of size dispersion, whereas others yielded quite large aggregates, while in toluene mostly highly aggregated particles were observed. It is also worth noting that nanoparticles after hydrolysis **3a**–**j** gave the best results in acetone. It is not possible to draw any meaningful size-dispersion—structure relationship for these species from these results. In Table 1 all DLS results in four solvents are summarized. 

**Figure 6:**
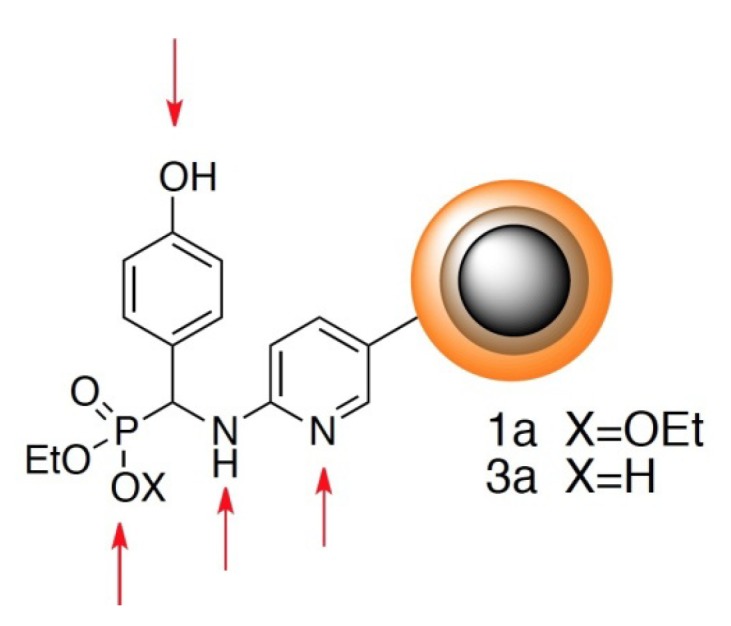
Representative structure of nanoparticle—arrows indicate protonation sites.

The particles obtained without the application of linker are characterized by an enormous dispersion of size in water and therefore we decided to modify their structure by the application of linkers. The latter structures (a representative one is shown in Figure 6) contain multiple protonation sites, which means that they also tend to aggregate in a pH-dependent manner. They may be considered as polyionic surfactant-like species. 

**Table 1:** DLS results in four solvents.

No	Hydrodynamic size [nm] (fraction %)			
	Water	Methanol	Acetone	Toluene
**1a**	168 (79%), 954 (21%)	144 (100%)	**85** (100%)	Above 1 µm
**1b**	710 (100%)	184 (100%)	399 (100%)	507 (10%)
**1c**	485 (100%)	**72 (100%)**	39 (49%), **81(51%)**	194 (36%), 808 (38%)
**1d**	**74 (47%)**, 259 (53%)	180 (100%)	**96 (100%)**	280 (23%), 870 (77%)
**1e**	180 (91%), 840 (8%)	170 (100%)	121 (100%)	697 (100%)
**1f**	820 (100%)	**70 (40%)**, 337 (60%)	**73 (40%)**, 280 (60%)	560 (40%)
**1g**	402 (25%), 930 (75%)	159 (100%)	294 (100%)	Above 1 µm
**1i**	296 (100%)	**65 (100%)**	114 (100%)	495 (100%)
**1j**	519 (100%)	139 (100%)	294 (100%)	Above 1 µm
**2d**	177 (28%)	202 (100%)	Powyżej 1 µm	Above 1 µm
**2g**	36 (15%), 116 (85%)	178 (100%)	**95 (100%)**	Above 1 µm
**3a**	105 (100%)	120 (78%)	**86 (100%)**	Above 1 µm
**3c**	**100 (39%)**, 319 (61%)	130 (100%)	**82 (100%)**	349 (100%)
**3d**	**93 (38%)**, 277 (62%)	109 (100%)	**77 (100%)**	Above 1 µm
**3e**	158 (15%), 406 (85%)	**82 (22%)**, 125 (78%)	**96 (100%)**	377 (58%), 840 (42%)
**3j**	630 (100%)	209 (100%)	**86 (100%)**	635 (100%)
**4d**	602 (100%)	196 (100%)	**80 (100%)**	249 (46%), 748(54%)
**4g**	128 (52%), 380 (48%)	150 (100%)	198 (100%)	175 (100%)
**5b**	490 (30%), 770 (70%)	**71 (100%)**	**86 (100%)**	215 (25%), 743 (75%)
**5g**	123 (100%)	130 (18%), 346 (82%)	**93 (100%)**	Above 1 µm
**5h**	105 (100%)	118 (100%)	160 (100%)	222 (100%)

**Figure 7:**
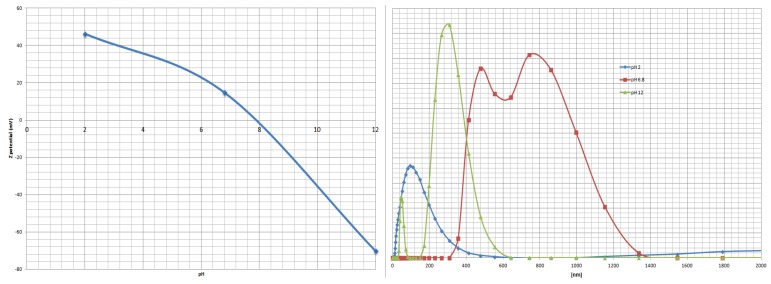
Size distribution of nanoparticles **2g** in different pH values.

Nanoparticles (both esters and acids) gave satisfactory size-dispersion when directly suspended in water, being usually in the range of 100 nm. The average sizes of the obtained particles were, however, usually higher that those obtained in methanol. Therefore we had measured their dependence on the pH of the aqueous solution by applying pH of 2, 6.8 and 12. At neutral pH, a strong tendency to aggregation was observed. A representative example obtained is shown in Figure 7. As expected, a significant reduction of the average size and size dispersion was obtained in strongly basic (pH 12) solutions, where all the residues are unprotonated. Also no aggregation was observed at this pH. More complex, and dependent on the chemical structure of aminophosphonate side chain, is the influence of acidic pH. In the cases when the aminophosphonate side chain is hydrophobic it usually has a positive influence on nanoparticle dispersion. Also in this case it is difficult to draw any reasonable structure-behavior relationship. 

The stability of nanoparticles dispersions correlates well with the ζ−potential measurements. It is generally accepted that the potential higher than 30 meV or lower than −30 meV characterizes stable systems. This is also the case in these studies. 

## 3. Experimental

### 3.1. General

All solvents and reagents were purchased from commercial suppliers (Aldrich, Sigma, Merck, POCh), were of analytical grade and were used without further purification. Infrared spectra were measured on a 1600 FT-IR Perkin-Elmer spectrometer. The size of the nanoparticles was determined by dynamic light scattering using a Malvern Zetasizer Nano ZS in Laboratory of Biomedical Chemistry, Department of Experimental Oncology, Institute of Immunology and Experimental Therapy, PAS, Wroclaw. NMR experiments were performed on a Bruker DRX AVANCE TM 300MHz spectrometer. SEM-EDX and TEM analyses were performed in the Microscopy and Analysis Suite, University of Bath, on a JEOL JSM6480LV with an Oxford INCA X-ray analyzer and an OEL JEM 1200EXII, respectively. 

### 3.2. The Synthesis of Iron Oxide Nanoparticles Coated with Silica

To a one-litre three neck flask of water (188 mL) and 23% aqueous solution of ammonia (10 mL) was added. The solution was stirred under a nitrogen atmosphere. At the same time a mixture of FeCl_3_·6H_2_O and FeCl_2_·4H_2_O (in a molar ratio of 1:2) was dissolved in water (10 mL) containing 36% HCl solution (0.125 mL). This solution of chloride salts was very slowly added dropwise to the solution of NH_4_OH with vigorous stirring. A black precipitate of iron oxide nanoparticles was formed and isolated through magnetic decantation. Magnetic nanoparticles were washed several times with distilled water and finally with 0.1 M solution of tetramethylammoniun hydroxide (TMAOH, 100 mL). This mixture was sonicated for 1 h, and the nanoparticles were isolated through magnetic decantation and dried. 

Iron oxide nanoparticles (0.1 g) were placed in distilled water (100 mL) and sonicated for 1 h. Then 2-propanol (100 mL) was added and the mixture sonicated for an additional 3 h. After that 23% aqueous solution of ammonia (1 mL) was added. The mixture was vigorously stirred and tetraethylortosilicate (0.2 mL) was added. After 2 h of stirring the nanoparticles were separated through magnetic decantation, washed with water and dried.

### 3.3. The Functionalization of Silica Coated Nanoparticles with (3-aminopropyl)trimethoxysilane

Freshly coated iron oxide nanoparticles (0.5 g), water (25 mL) and methanol (25 mL) were placed in a flask and sonicated for 1 h. After this time (3-aminepropyl)trimethoxylsilane (0.75 mL) was added in one portion and the mixture stirred at 80 °C for 5 h. The brown precipitate was isolated through magnetic decantation, washed with water and dried. 

### 3.4. The General Procedure Followed for the Synthesis of Aminophosphonate-Functionalized Magnetic Nanoparticles

Nanoparticles functionalized surfacially with amino-groups (0.5 g) were mixed with 2-amino-5-chloropyridine (0.5 g) or 2,6-diamino-4-chloropyrimidine (0.5 g) in toluene and refluxed for 8h. After this time, the solvent was evaporated, the precipitate washed with fresh toluene and magnetically decanted. The synthesised nanoparticles were then used as amine reagent for the synthesis of aminophosphonates. Thus, the appropriate amount of nanoparticles (0.5 g) and aldehyde (1 g) were mixed in toluene and refluxed until the amount of collected water remained unchanged in the Dean-Stark trap. The solvent was evaporated and the crude nanoparticle Schiff base was used in the next step without purification. It was suspended in a new portion of toluene and the equivalent of diethyl phosphite (in 1:1 molar ratio to aldehyde) was added followed by refluxing for 4 h. The solvent was then evaporated, the residue washed with toluene, and water and isolated through magnetic decantation. 

**1a**: IR (KBr, cm^−1^): 570 (m, Fe-O), 1049 (s, P-O-C), 1093 (s, Si-O), 1176 (s, Si-C), 1218 (s, P=O), 1479 (s, N-H), 1596 (s, C_Ar_-N_Ar_), 2919, 2990 (m, C-H), 3326 (m, Ar-O-H); SEM-EDX (KeV): 0.1 (C), 0.5 (O), 1.9 (Si), 2.0 (P), 6.7 (Fe).

**1b**: IR (KBr, cm^−1^): 569.8 (m, Fe-O), 1037 (s, P-O-C), 1060 (s, Si-O), 1172 (s, Si-C), 1284 (m, P=O), 1479 (s, N-H), 1596 (s, C_Ar_-N_Ar_), 2919 (m, C-H); SEM-EDX (KeV): 0.1 (C), 0.5 (O), 1.9 (Si), 2.0 (P), 6.7 (Fe).

**1c**: IR (KBr, cm^−1^): 541 (m, Fe-O), 1020 (s, P-O-C), 1048 (s, Si-O), 1230 (s, P=O), 1478 (s, N-H), 1598 (s, C_Ar_-N_Ar_), 2899, (m, C-H); SEM-EDX (KeV): 0.1 (C), 0.5 (O), 1.9 (Si), 2.0 (P), 6.7 (Fe). 

**1d**: IR (KBr, cm^−1^): 567 (m, Fe-O), 1029 (s, P-O-C), 1054 (s, Si-O), 1230 (s, P=O), 1247 (s, Si-C), 1484 (s, N-H), 1598 (s, C_Ar_-N_Ar_), 2865 (m, C-H), 3285,14 (s, N-H); SEM-EDX (KeV): 0.1 (C), 0.5 (O), 1.9 (Si), 2.0 (P), 6.7 (Fe). 

**1e**: IR: (KBr, cm^−1^): 577 (m, Fe-O), 1022 (s, P-O-C), 1053 (s, Si-O), 1475 (s, N-H), 1598 (s, C_Ar_-N_Ar_), 2906 (m, C-H), 3294 (m, N-H); SEM-EDX (KeV): 0.1 (C), 0.5 (O), 1.9 (Si), 2.0 (P), 6.7 (Fe). 

**1f**: IR: (KBr, cm^−1^): 533 (m, Fe-O), 1036 (s, P-O-C), 1224 (s, P-O), 1288 (m, Si-C), 1481 (s, N-H), 1596 (s, C_Ar_-N_Ar_), 2901, 2979 (m, C-H), 3283 (s, N-H); SEM-EDX (KeV): 0.1 (C), 0.5 (O), 1.9 (Si), 2.0 (P), 6.7 (Fe). 

**1g**: IR (KBr, cm^−1^): 583 (m, Fe-O), 1032 (s, P-O-C), 1052 (m, Si-C), 1238 (s, P=O), 1369 (m, N-O), 1481 (m, N-H), 1533 (m, C_Ar_-N_Ar_), 2976 (m, C-H), 3307 (s, N-H); SEM-EDX (KeV): 0.1 (C), 0.5 (O), 1.9 (Si), 2.0 (P), 6.7 (Fe). 

**1h**: IR (KBr, cm^−1^): 583 (m, Fe-O), 1028 (s, P-O-C), 1028 (m, Si-C), 1238 (s, P=O), 1354 (m, N-O), 1499 (m, N-H), 1533 (m, C_Ar_-N_Ar_), 2986 (m, C-H), 3419 (s, N-H); SEM-EDX (KeV): 0.1 (C), 0.5 (O), 1.9 (Si), 2.0 (P), 6.7 (Fe).

**1i**: IR (KBr, cm^−1^): 568 (m, Fe-O), 1045 (s, P-O-C), 1162 (m, Si-O), 1224 (m, P=O), 1336 (m, N-O), 1439 (m, N-H), 1508 (m, C_Ar_-N_Ar_), 2982 (m, C-H), 3413 (s, N-H); SEM-EDX (KeV): 0.1 (C), 0.5 (O), 1.9 (Si), 2.0 (P), 6.7 (Fe).

**1j**: IR (KBr, cm^−1^): 571 (m, Fe-O), 1096 (s, P-O-C), 1148 (s, Si-O), 1213 (s, P-O), 1246 (s, Si-C), 1459 (m, N-H), 2832, 2938 (m, C-H), 3187 (m, Ar-O-H), 3240 (s, N-H); SEM-EDX (KeV): 0.1 (C), 0.5 (O), 1.9 (Si), 2.0 (P), 6.7 (Fe).

**2d**: IR (KBr, cm^−1^): 560 (m, Fe-O), 1051 (s, P-O-C), 1162 (s, Si-O), 1495 (m, N-H), 1579 (s, C_Ar_-N_Ar_), 2931, 2982, (m, C-H), 3399 (s, N-H); SEM-EDX (KeV): 0.1 (C), 0.5 (O), 1.9 (Si), 2.0 (P), 6.7 (Fe).

**2g**: IR (KBr, cm^−1^): 546 (m, Fe-O), 1049 (s, P-O-C), 1162 (m, Si-O, Si-C), 1296 (m, Ar-O-C), 1467 (s, N-H), 1579 (s, C_Ar_-N_Ar_), 2980 (m, C-H), 3100 (m, Ar-O-H), 3303 (s, N-H); SEM-EDX (KeV): 0.1 (C), 0.5 (O), 1.9 (Si), 2.0 (P), 6.7 (Fe).

### 3.5. The General Synthesis of Monoesters

The nanoparticle diester (0.5 g) and the sodium iodide (0.5 g) were placed in acetone and refluxed for 3 h. The sodium salt of the monoester was isolated through magnetic decantation, washed with acetone and converted into acid form by washing with 1 M HCl. The obtained nanoparticles were washed with water and dried.

**3a**: R (KBr, cm^−1^): 577 (m, Fe-O), 1050 (s, P-O-C), 1068 (s, Si-O), 1189 (s, Si-C), 1223 (m, P=O), 1607 (s, N-H), 1656 (s, C_Ar_-N_Ar_), 2737 (m, C-H), 3257 (m, AO-H), 3417 (s, N-H).

**3b**: IR (KBr, cm^−1^): 570 (m, Fe-O), 1037 (s, P-O-C), 1060 (s, Si-O), 1173 (s, Si-C), 1284 (m, P=O), 1479 (s, N-H), 1596 (s, C_Ar_-N_Ar_), 2919, 3228 (m, C-H).

**3c**: IR (KBr, cm^−1^): 541 (m, Fe-O), 1012 (s, P-O-C), 1044 (s, Si-O), 1212 (s, P=O), 1473 (s, N-H), 2849, 2917 (s, C-H), 3429 (m, O-H).

**3d**: IR (KBr, cm^−1^): 569 (m, Fe-O), 1095 (s, P-O-C), 1228 (s, P=O), 1482 (s, N-H), 1598 (s, C_Ar_-N_Ar_), 3469 (s, O-H).

**3e**: IR (KBr, cm^−1^): 611 (m, Fe-O), 1027 (m, P-O-C), 1054 (m, Si-O), 1479 (m, N-H), 1598 (m, C_Ar_-N_Ar_), 3416 (s, O-H).

**3e**: IR (KBr, cm^−1^): 611 (m, Fe-O), 1027 (m, P-O-C), 1054 (m, Si-O), 1479 (m, N-H), 1598 (m, C_Ar_-N_Ar_), 3416 (s, O-H).

**3f**: IR: (KBr, cm^−1^): 536 (m, Fe-O), 1026 (s, P-O-C), 1052 (s, Si-O), 1228 (m, P=O), 1478 (s, N-H), 1597 (s, C_Ar_-N_Ar_), 2984, (m, C-H), 3468 (s, O-H).

**3g**: IR (KBr, cm^−1^): 546 (m, Fe-O), 1024 (m, P-O-C), 1431 (w, N-H), 1605 (m, C_Ar_-N_Ar_), 3398 (s, O-H).

**3h**: IR (KBr, cm^−1^): 611 (m, Fe-O), 1044 (m, P-O-C), 1162 (M, Si-O, Si-C), 1239 (m, P=O), 1355 (m, N-O), 1499 (w, N-H), 3416 (s, N-H), 3468 (s, O-H).

**3i**: R (KBr, cm^−1^): 570 (m, Fe-O), 1047 (m, P-O-C), 1162 (m, Si-O), 1227 (m, P=O), 1336 (m, N-O), 1440 (w, N-H), 1510 (m, C_Ar_-N_Ar_), 3415 (s, N-H), 3471 (s, O-H).

**3j**: IR (KBr, cm^−1^): 571 (m, Fe-O), 1096 (s, P-O-C), 1148 (s, Si-O), 1213 (s, P=O), 1460 (m, N-H), 1625 (s, C_Ar_-N_Ar_), 2832, 2938 (m, C-H), 3187 (m, Ar-O-H), 3240 (m, N-H), 3412 (m, O-H).

## 4. Conclusions

Hydrophosphonylation surfacial Schiff bases obtained from iron oxide magnetic nanoparticles functionalized with amino groups appeared to be an effective method for preparation of aminophosphonate-functionalized nanoparticles. By being surfacially charged (both negative and positive) these particles behave as micelles.
